# Myopathology of Adult and Paediatric Mitochondrial Diseases

**DOI:** 10.3390/jcm6070064

**Published:** 2017-07-04

**Authors:** Rahul Phadke

**Affiliations:** 1 Division of Neuropathology, UCL Institute of Neurology, National Hospital for Neurology and Neurosurgery, UCLH NHS Foundation Trust, London WC1N 3BG, UK; r.phadke@ucl.ac.uk; Tel.: +44-020-344-84393; 2 Dubowitz Neuromuscular Centre, Great Ormond Street Hospital for Children NHS Foundation Trust, London WC1N 3JH, UK

**Keywords:** mitochondrial, muscle biopsy, ragged red, COX-negative, subsarcolemmal, immunohistochemistry

## Abstract

Mitochondria are dynamic organelles ubiquitously present in nucleated eukaryotic cells, subserving multiple metabolic functions, including cellular ATP generation by oxidative phosphorylation (OXPHOS). The OXPHOS machinery comprises five transmembrane respiratory chain enzyme complexes (RC). Defective OXPHOS gives rise to mitochondrial diseases (mtD). The incredible phenotypic and genetic diversity of mtD can be attributed at least in part to the RC dual genetic control (nuclear DNA (nDNA) and mitochondrial DNA (mtDNA)) and the complex interaction between the two genomes. Despite the increasing use of next-generation-sequencing (NGS) and various omics platforms in unravelling novel mtD genes and pathomechanisms, current clinical practice for investigating mtD essentially involves a multipronged approach including clinical assessment, metabolic screening, imaging, pathological, biochemical and functional testing to guide molecular genetic analysis. This review addresses the broad muscle pathology landscape including genotype–phenotype correlations in adult and paediatric mtD, the role of immunodiagnostics in understanding some of the pathomechanisms underpinning the canonical features of mtD, and recent diagnostic advances in the field.

## 1. Introduction

The diagnosis of mtD is challenging due to the incredible phenotypic and genetic diversity associated with these diseases. This partly stems from the dual genetic control (nDNA and mtDNA) of the RC, the complexity of intergenomic signalling and its functional consequences. mtD can be inherited in an autosomal dominant, autosomal recessive, X-linked or mitochondrial (i.e., maternal) fashion. The circular mtDNA encodes 13 RC subunits, 22 mitochondrial tRNAs and two ribosomal RNAs. Additionally, the mitoproteome requires over 1300 nuclear encoded proteins to produce, assemble and support the five multimeric OXPHOS RC (I–V), and ancillary mitochondrial processes [1,2,3]. Tissues and organs affected in mtD are often those with high-energy requirements. Clinical symptoms can manifest at any age, and can affect a single organ or be multisystemic [4]. Typically, the more severe phenotypes present early, and milder phenotypes present later in life [5]. There are classic clinical syndromes with stereotypic features such as Leigh syndrome (subacute necrotising encephalomyopathy), MELAS (mitochondrial myopathy, encephalopathy, lactic acidosis and stroke-like episodes) and Alpers disease (epilepsy and liver failure). Point mutations and large-scale mtDNA deletions represent the two most common causes of primary mtDNA disease, the former usually being maternally inherited, and the latter typically arising de novo during embryonic development [1]. Exemplary genotype–phenotype associations are recognised, often in a syndromic context, e.g., mitochondrial protein synthesis *tRNA* gene point mutations are associated with MELAS, myoclonic epilepsy with ragged red fibres (MERRF) and syndromic forms of maternally transmitted diabetes; mutations in mitochondrial RC protein coding genes are associated with Leber hereditary optic neuropathy (LHON), neuropathy, ataxia, retinitis pigmentosa (NARP), and maternally inherited Leigh syndrome (MILS); single mtDNA deletions are associated with Pearson syndrome, chronic progressive external ophthalmoplegia (CPEO) and Kearns-Sayre syndrome (KSS); multiple mtDNA deletions are associated with mitochondrial neurogastrointestinal encephalomyopathy (MNGIE), autosomal dominant or autosomal recessive progressive external ophthalmoplegia (AD/AR-PEO), and sensory ataxic neuropathy, ataxia, ophthalmoplegia (SANDO); mtDNA depletion are associated with early-onset myopathic and hepatocerebral forms; Leigh syndrome is associated with a variable genotypic background including nDNA and mtDNA mutations; and so on [6,7]. However, many patients display non-specific features of developmental delay or regression, further hindering accurate diagnosis [8]. The onset of symptoms, phenotypic variability, and variable penetrance of mtD are influenced by the peculiarities of mitochondrial genetics including the threshold effect, mitotic segregation, clonal expansion and a genetic bottleneck, as well as the nuclear genome background in which it coexists or by environmental and epigenetic factors [6]. 

## 2. Laboratory Investigations and the Rationale for Muscle Biopsy

Given the complexity of mtD phenotypes and genetics, securing a diagnosis frequently requires extensive non-invasive and invasive tests including imaging, neurophysiology, metabolic and biochemical studies, muscle pathology and functional testing, followed by definitive molecular genetic confirmation. Resting and exercise induced increase in blood lactate is a useful albeit non-specific screening tool for mtD, but can be normal or minimally elevated as in mitochondrial polymerase gamma (POLG1) associated diseases, Leber Hereditary Optic Neuropathy (LHON), Leigh disease, Kearns-Sayre syndrome and Complex I deficiency [9]. The blood lactate/pyruvate ratio may increase in inborn errors of the mitochondrial respiratory chain [10]. Spurious elevation of plasma lactate and/or pyruvate may occur from poor collection or handling technique, secondary mitochondrial dysfunction in a range of systemic and metabolic diseases, and in nutritional thiamine deficiency. Blood and/or CSF pyruvate levels may increase in defects of pyruvate metabolism. Similarly, CSF lactate and/or pyruvate levels may increase without blood elevation in mtD with predominant CNS manifestations [11]. Elevated plasma/CSF amino acids, urine organic acids, and plasma acylcarnitines all suggest underlying mitochondrial dysfunction, however, normal levels do not exclude mtD [12,13]. CK values are normal or mildly elevated, unless measured in the setting of rhabdomyolysis [14]. Neurophysiology may show non-specific signs of a myopathy, or a neuropathy when present, but may be normal. Neuroimaging, encompassing several modalities beyond routine T1 and T2 magnetic resonance imaging (MRI), including volumetric analysis, diffuse tensor imaging (DTI), magnetic resonance spectroscopy (MRS), arterial spin labelling, and functional magnetic resonance imaging (fMRI) has shown its potential as an investigative tool, and in many cases, providing non-invasive and repeatable biomarker inquiry in patients with mtD [15]. MRI findings in patients with mtD can often be non-specific, including in those with clinical central nervous system involvement; however, it is the pattern of involvement that can suggest an underlying neurometabolic defect. In children, a common pattern is “global” delay in myelination early in the course of the disease [16]. The most common specific MRI findings are a symmetrical signal abnormality of deep grey matter presenting with hyperintensity on T2 and FLAIR images, and hypointensity on T1 images. Any deep structure can be involved and the character of the lesion can be either patchy or homogeneous. Cerebral and cerebellar atrophy may be present in varying degrees. These specific MRI findings are more likely to be associated with well recognised syndromic phenotypes such as Leigh disease, MERRF, MELAS, KSS, MNGIE, etc. [17]. MRS can provide valuable in vivo metabolic information to measure metabolites possessing resonating nuclei (hydrogen-1; _1_H: phosphorous-31; _31_P: carbon-13; _13_C) in the mM range. mtD represent a particularly prominent set of diseases that show MRS changes due to the consequences of impaired OXPHOS. The most consistent MRS change accompanying increased lactate in mitochondrial disease is decreased *N*-acetyl-l-aspartate (NAA) normalised to creatine, suggestive of cellular compromise [12,17]. Other brain metabolites such as myo-inositol, choline, creatinine and succinate can be measured by MRS. One disease biomarker that appears highly specific to complex II disease is a large elevation in succinate in white matter [18]. MRS is being increasingly applied as a non-invasive tool to monitor the effects of therapeutic intervention in patients with mtD. Diagnostically useful patterns of selective muscle involvement are well-recognised and increasingly being used in the diagnostic algorithm for muscular dystrophies, congenital myopathies and a few other heritable neuromuscular disorders [19]. This approach appears less useful in mtD, probably reflecting the fact that clinically and biochemically affected muscles in mtD rarely ever show significant fibro-fatty infiltration when examined histologically.

There is no single “gold standard” laboratory test for diagnosing mtD. The screening tests described above broadly confirm presence of dysfunction in various organ systems and help to increase or decrease the clinical suspicion of mtD. More invasive testing is necessary to establish direct morphological, biochemical and molecular genetic evidence of mitochondrial dysfunction [5]. In principle, the relevant tissue to investigate is one that clinically expresses disease. Skeletal muscle remains the tissue of choice, and is frequently sampled in part due to the relative safety and ease with which tissue samples can be obtained. It can provide valuable diagnostic information in many cases, even without clinically overt myopathic involvement [20,21,22]. Skeletal muscle is a post-mitotic terminally differentiated tissue with only limited regenerative capacity via satellite cell transformation. This terminal differentiation results in a fairly stable lifelong relationship between the mutant and wild-type mtDNA ratio (heteroplasmy) in contrast to nucleated blood cells in which this ratio can decrease due to selection pressure, thereby obscuring evidence of mitochondrial dysfunction [22]. Skeletal muscle mitochondria are abundant in subsarcolemmal and intermyofibrillar locations and larger than in most other tissues. Pathological assessment of “non-muscle” components in biopsies including blood vessels and nerves can provide evidence of multi-organ dysfunction [21]. Biochemical testing of respiratory chain enzyme dysfunction typically involves determination of individual or paired respiratory chain enzyme complex activities in mitochondrial fractions or tissue homogenates prepared from fresh or frozen muscle tissue. Biochemical assays have low inter-laboratory reproducibility and a systematic program to share samples and standardise methodologies across diagnostic laboratories has not been implemented. Other confounding factors include masking of a RC defect in tissue homogenates due to low-level heteroplasmy and a physiological compensatory mitochondrial proliferative response [8]. In this context, parallel histological assessment of skeletal muscle can uniquely provide histochemical evidence of RC defect at the single cell level. Simultaneously, the biopsy can be assessed for a number of conditions in the clinical differential diagnosis that can mimic a mitochondrial myopathy or induce secondary mitochondrial dysfunction. This includes fatty acid oxidation defects, glycogen storage disorders, endocrine, congenital and inflammatory myopathies and muscular dystrophies. The reliability of detecting morphological and histochemical abnormalities in skeletal muscle in mtD has led to their inclusion as major and minor criteria in several classification schemes for diagnosing mtD in adults and children [23,24,25,26]. It is standard practice to perform a skin biopsy in parallel to a muscle biopsy primarily for establishing fibroblast cultures. While it is less invasive, it is not uncommon for patients with OXPHOS defects in skeletal muscle to have normal RC activities in fibroblasts [27,28]. This in part due to altered heteroplasmy and high tissue regeneration rate of fibroblasts compared to skeletal muscle [27]. It is equally important to recognise the limitations of muscle biopsy analysis in investigating mtD. RC deficiencies are usually tissue specific, particularly if sporadic and somatic, further influenced by the type of mutation and the peculiarities of mitochondrial genetics. Therefore even muscle samples with proven mtD mutations/phenotypes may not show pathological and/or biochemical evidence of mitochondrial dysfunction [14]. mtDNA copy number analysis in muscle tissue by real time qPCR normalised to age-matched controls gives an indication of depletion or amplification of mtDNA content. mtDNA depletion can point towards mtDNA depletion syndromes caused by a number of genes involved in mtDNA maintenance. mtDNA depletion in muscle is however not as obvious in patients with the myopathic disease form as it is in liver tissue in patients with the hepatocerebral form [29]. There are no reliable histochemical assays for demonstration of defects in complexes I, III and V [1,21]. Muscle biopsy may appear histologically normal even in the context of genetically confirmed mtD and when the biochemical defect does not involve complex IV [23,30]. The histological interpretation of paediatric muscle biopsies can be challenging in the absence of age-matched controls. Morphological and histochemical abnormalities of mitochondria are not entirely specific and are seen secondary to other myopathic processes and with ageing. Despite its invasive nature and limitations, muscle biopsy remains the gold standard for mtD, especially due to primary mtDNA mutations [31].

## 3. Technical Considerations

Muscle and skin biopsies must be performed and processed in a manner that optimally preserves mitochondrial morphology, enzymatic/functional activity, protein and DNA/RNA content to allow for the broadest range of tissue investigations into mitochondrial dysfunction. This requires good communication between clinicians, surgeons and pathologists and teamwork. Rigorous implementation of a standardised biopsy protocol minimises the risk of ambiguity in interpretation of results due to a myriad of artefacts resulting from improper sampling and processing. Some of these issues are discussed below. A limb muscle such as vastus lateralis, gastrocnemius, deltoid or biceps brachii is selected for sampling, depending on the institutional preference. Occasionally extraocular muscles may be sampled in patients with external ophthalmoplegia. However, these muscles may normally harbour features considered “myopathic” for limb muscles, and show a greater prevalence of ragged red fibres (RRF) and COX-negative fibres compared to limb muscles from the third decade of life [32,33]. Skeletal muscle can be harvested via an open biopsy or a needle biopsy procedure. The latter has the advantage of being performed under local anaesthesia and/or deep sedation, and producing a smaller scar. Concerns about tissue fragmentation and smaller tissue yield that may be insufficient for biochemical assays have been addressed by implementing protocols using the modified Bergström needle for sampling. Ideally, the sample should be examined under a dissecting microscope in the procedure room for adequacy and orientation. A portion of fresh unfixed muscle is immediately placed in RNase free tubes and snap frozen in liquid nitrogen for biochemistry and genetic testing. The best oriented portion can be transported to the laboratory wrapped in cling film or by placing in a closed petri dish on a piece of gauze lightly moistened in saline, and then frozen in isopentane cooled in liquid nitrogen for histology, histochemistry and immunohistochemistry. A small longitudinal piece 0.5 mm long is placed in chilled 2% glutaraldehyde for electron microscopy. Functional assays including polarographic studies require fresh, unfixed tissue. Excessive mechanical trauma to the sampled tissue, infiltration of the local aesthetic into the fascicles, drying out and excess contact with saline can render morphology and histochemistry uninterpretable. Isopentane may interfere with measurement of complexes I, II and III in biochemical assays and give falsely low activities [34]. Many laboratories routinely fix a portion of muscle in 10% formalin for paraffin embedding. Apart from providing a larger sampling field and with the exception of high-risk infectious samples, this practice has several disadvantages and its routine implementation should be discouraged. Formalin fixation causes loss of histochemical enzyme activity, yields inferior muscle morphology, enhances autofluorescence, and may require laborious antigen retrieval protocols for protein immunohistochemistry. In case of large open biopsies, an additional block can be prepared for freezing in isopentane, and any surplus tissue can be snap frozen in liquid nitrogen. A 4 × 4 mm skin punch biopsy from the thigh provides sufficient tissue for growing fibroblast cultures and should be placed in sterile conditions in a culture medium containing uridine and pyruvate to prevent the loss of cells harbouring mutant mtDNA [35]. Detailed protocols for sampling and processing of skin and muscle biopsies have been published [22,36].

## 4. Histochemical Assays for Detecting RC Defects

The mitochondrial RC responsible for cellular ATP generation is located on the inner mitochondrial membrane comprising five complexes: CI, NADH-coenzyme Q reductase; CII, succinate-CoQ reductase that includes the FAD-dependent succinate dehydrogenase (SDH) and iron-sulphur proteins; CIII, reduced CoQ-cytochrome C reductase; CIV, cytochrome C oxidase; and CV, ATP synthase [37]. The free energy generated via redox reactions involving electron transfer across the complexes to molecular oxygen creates a transmembrane proton gradient—protons are pumped through CI, CIII and CIV, and CV allows protons to flow back into the mitochondrial matrix, and the released energy is used to synthesise ATP. Histochemical stains are available that can demonstrate activities of CI (NADH-TR), CII (SDH) and CIV (COX). NADH-TR stain comprises reduced nicotinamide adenine dinucleotide as the substrate that is oxidized by NADH-dependent enzymes. Addition of a tetrazolium salt (NBT) results in the deposition of a reduced, insoluble blue formazan product at the reaction site. TR denotes tetrazolium reductase. However, the NADH is not only oxidized by CI of the RC, but also sarcoplasmic reticulum (SR) NADH-oxidising enzymes. In the absence of specific inhibitors of the “non-mitochondrial” NADH-oxidase activity, this stain is not specific for CI, and any CI defect is invariably masked [38]. The advantage is this stain can be used as a general marker of mitochondria and SR, and thereby is excellent in highlighting structural defects such as cores or mini-cores. SDH stain can demonstrate CII activity. Na-succinate is used as the substrate, which gets oxidized to fumarate by CII in the presence of NBT, which is reduced to insoluble blue formazan at the reaction site [38]. In the modified SDH reaction, 1-methoxyphenazine methosulphate (mPMS) or phenazine methosulphate (PMS) are added to the incubation medium as exogenous electron carriers and azide or cyanide as inhibitors of cytochrome oxidase. The mPMS and azide substitution results in substantial reduction in the non-specific reduction of NBT and a linear reaction rate, thereby allowing better histochemical quantitation [39]. Nuclear genes encode all sub-units of CII; therefore, CII is rarely affected in diseases with primary mtDNA defects. COX stain demonstrates cytochrome C oxidase activity. In this reaction, diaminobenzidine (DAB) acts as the electron donor to reduce cytochrome C. The haeme units of CIV catalyse the transfer of electrons from reduced cytochrome C to molecular oxygen to form water. In turn, the oxidized DAB forms a brown coloured indamine polymer that is deposited at the reaction site. As continuous reoxidation of cytochrome C is required for the accumulation of the visible oxidized DAB, the reaction serves to visualize CIV activity [40]. Addition of catalase prevents contamination from endogenous peroxidase activity. In keeping with their physiological properties, in skeletal muscle, type I fibres show the darkest staining, type IIA fibres intermediate staining and type IIB (IIX) fibres show the weakest staining for all three reactions, commensurate with mitochondrial enrichment in these fibres. As the SDH and COX reactions specifically demonstrate CII and CIV activities, they are regarded as specific mitochondrial markers, and the intensity and distribution of staining allows simultaneous assessment of complex activity, mitochondrial mass and distribution at the single cell level as well as within a spatial two-dimensional context of the section. A further refinement in technique is the development of the sequential COX-SDH reaction, with early studies dating back to 1968, and now widely regarded as the optimal technique for demonstrating CIV defects, particularly in instances of heteroplasmic mtDNA defects. The technique relies on the preserved activity of CII (being entirely nDNA encoded) in cells with mtDNA defects. In cells with functional CIV, the brown indamine polymer product will localize in and saturate mitochondrial cristae. Those cells with reduced or absent CIV activity will not be saturated by the brown indamine polymer product, allowing for visualization of CII activity by deposition of the blue formazan end product [41,42,43]. Thus, in a normal skeletal muscle cross-section, the brown COX staining overshadows the blue SDH staining in all three fibre types. However, CIV deficient fibres will stand out as varying intensities of blue, depending on the deficiency being partial or complete. A number of variations in protocols for these histochemical reactions exist, and no attempt has been made yet to standardize protocols, at least amongst larger reference laboratories. Furthermore, these tests are highly susceptible to a variety of artefacts arising from poor sampling and processing, contributing to ambiguous results, or worse, false-positives and false-negatives. Excessive trauma to fibres, excess saline contact, drying out, repeated cycles of freeze-thawing, and drawing the hydrophobic barrier pen too close to the section can cause loss of activity. Hypercontracted fibres can cause fibres to appear darker. The sequential COX-SDH reaction should not be substituted for, but rather be run in parallel with individual COX and SDH reactions. Several quality control measures to preserve the integrity of the COX-SDH reaction have been outlined [44]. 

## 5. Canonical Pathological Features 

Pathological changes in skeletal muscle biopsies from individuals with mtD can be varied, depending on the underlying genotype. Although not entirely specific, ragged red fibres (RRF) and COX-negative fibres are widely regarded to be the canonical features of mtD pathology [6,7,12,21,22,26,45,46,47,48]. Another useful diagnostic feature is SDH deficiency, although rare. 

### 5.1. Ragged Red Fibres (RRF)

The recognition of RRF as a morphological hallmark of mtD predates the molecular era. Mitochondrial myopathies were described in the 1960s when systematic histochemical and ultrastructural studies revealed excessive proliferation of normal or abnormal-looking mitochondria in skeletal muscle of patients with weakness or exercise intolerance [30,49,50]. With the development of the modified Gomori Trichrome (MGT) stain allowing visualisation of connective tissue (light green), nuclei (red/purple), mitochondria, sarcoplasmic reticulum, sarcolemma (red) and myofibrils (green) in frozen sections, the abnormal fibres in these conditions showed up as bright red accumulation of staining and “cracking” of the fibre edges, corresponding to the massive irregular proliferation of mitochondria, and were dubbed “ragged red” [51]. The reason for the red staining is the affinity of chromotrome-2R, one of the MGT constituents that is lipophilic, and binds to sphingomyelin that is in abundance in mitochondrial membranes [21]. RRF usually show ultrastructurally abnormal mitochondria that frequently contain paracrystalline inclusions [47]. RRF are difficult to identify with MGT in formalin-fixed tissue as normal myofibrils stain red post-fixation. The red proliferative zones can be identified in Haematoxylin and Eosin-stained sections as subsarcolemmal areas of amorphous, basophilic staining [21]. SDH histochemistry shows increased staining in RRF, and such fibres appear as ragged blue fibres [22]. The term ragged red fibre equivalents (RRF equivalents) has been used to describe muscle fibres with mitochondrial accumulation showing positive staining with the modified SDH reaction, and the modified SDH reaction was demonstrated to be more sensitive than MGT in highlighting myofibres with increased mitochondrial proliferation [52]. RRF accumulate a very high percentage of mutant mitochondrial genomes >80% [53]. In longitudinal sections, RRF appear as segmental abnormalities, and there is a correlation between defective OXPHOS and the segmental abnormality suggesting that the abnormal proliferation is consequent to the defective OXPHOS [45,54]. RRF are seen in syndromic presentations of defects in mitochondrial protein synthesis (mtDNA rearrangements and point mutations), being more prevalent in MELAS, MERRF, KSS, and less frequently in CPEO [7,12]. RRF are usually absent in patients with syndromic presentations of defects in mitochondrial protein coding genes (LHON, NARP), however, few RRF may be seen in myopathic forms with isolated defects of CI, CIII and CIV due to mutations in mtDNA genes encoding ND subunits, cytochrome b, and COX subunits respectively [7]. In patients with mutations in nDNA genes encoding subunits or ancillary proteins of the RC, RRF are usually absent, e.g., in autosomal recessive Leigh syndrome due to mutations in CI or CII subunits, and Mendelian defects in assembly factors of CIV causing COX-deficient Leigh syndrome [7,30]. RRF are present in myopathic and encephalomyopathic forms of primary CoQ10 deficiency [55]. RRF are also present in myopathic forms of mtDNA depletion syndromes [56]. COX-negative (ragged blue) RRF are typically seen in MERRF, KSS and CPEO, when wild type mtDNA genomes fall below the threshold required to maintain CIV activity. In contrast, in classic MELAS due to the A3243G tRNA^Leu^ gene mutation, RRF are mostly COX-positive due to an even distribution of mutant and wild-type mtDNA genomes in these fibres [12,48,57,58]. RRF are COX-positive in mtDNA encoded CI and CIII subunit gene mutations, and COX-negative in CIV subunit gene mutations [7,12]. COX-negative RRF have also been observed in mtDNA depletion syndromes [59].

### 5.2. COX-Negative Fibres

A majority of individuals with mitochondrial myopathies that cause isolated or combined CIV deficiency due to mtDNA mutations harbour a mix of wild-type and mutant mtDNA molecules within each myofibre giving rise to heteroplasmy, a unique aspect of mitochondrial genetics. The proportion of mutant mtDNA can vary between individual myofibres [60,61]. A myofibre segment will develop biochemical OXPHOS deficiency when the mutant mtDNA exceeds a critical threshold, i.e., the level to which the cell can tolerate defective mtDNA molecules [62,63]. Heteroplasmic mutations have a variable threshold in different tissues. Furthermore, for unknown reasons, the threshold varies among mutation types and in skeletal muscle the mutation load for any particular tRNA (~90%) is typically higher than that for large-scale partial deletions of mtDNA (~70–80%) [61,64]. The threshold for mutation load in polypeptide-coding genes can be similarly broad, with low levels of mutation causing one type of clinical presentation and higher levels causing another, e.g., m.8993T→G mutation in the ATP synthase 6 (*ATP6*) gene: at mutation loads above 90%, manifests as maternally inherited Leigh’s syndrome (MILS), at mutation loads in the range of 70–90%, manifests with neuropathy, ataxia and retinitis pigmentosa (NARP), and by contrast, patients with 70% mutation in a tRNA will rarely display overt disease [48]. The consequent biochemical OXPHOS deficiency can be demonstrated histochemically in transverse sections of frozen skeletal muscle as a mosaic pattern of COX-positive and COX-negative fibres, which equally affect slow-twitch (oxidative) and fast-twitch (glycolytic) muscle fibres [63,65], and is considered a hallmark of mitochondrial disease [66]. In the stand-alone COX reaction, the COX-negative fibres appear unstained amongst the brown COX-positive fibres. In the SDH reaction, they often stain intense blue due to compensatory mitochondrial proliferation increasing the mitochondrial mass, and the fact that CII being entirely nuclear-encoded, its biochemical activity is usually intact in primary mtDNA defects. In the sequential COX-SDH reaction, COX-negative fibres appear blue amongst the brown COX-positive fibres. The broad genotypic correlations described above for RRF are also true for the presence of COX-negative fibres in mitochondrial myopathies. A mosaic pattern of COX-negative fibres is a robust marker of heteroplasmic mtDNA mutations affecting mitochondrial protein synthesis (rearrangements and point mutations in mitochondrial tRNA or ribosomal RNA genes), or rarely, affecting one of the three mtDNA encoded CIV sub-units [12,22,31]. A notable exception to this rule is a homoplasmic mutation in mitochondrial tRNA^Glu^ that is associated with a severe but reversible infantile mitochondrial myopathy and profound, though not exclusive, biochemical and histochemical COX deficiency, often accompanied by RRF in skeletal muscle [67,68]. The biopsy features in reversible and irreversible, fatal COX deficiency in the neonatal period are identical, and in both conditions the histochemical defect is restricted to extrafusal myofibres, sparing intrafusal muscle fibres and vascular smooth muscle [68,69,70,71]. Fatal infantile COX deficiency also affects the heart and brain, and has been linked to autosomal recessive mutations in COX assembly factors (*SCO2*, *COX15*, *COA5*, and *COA6*) [72,73,74,75]. Muscle biopsies from patients with defects in mtDNA maintenance will also show a similar mosaic COX-negative pattern due to overlapping influence of Mendelian and mitochondrial genetics, especially in cases of PEO with multiple mtDNA deletions [31,76,77]. In contrast, Mendelian disorders such as COX-deficient Leigh syndrome, e.g., due to mutations in COX assembly factors such as *SURF1*, will show diffuse COX-deficiency [78,79,80]. A mosaic COX deficiency is also found in cardiac muscle [81,82], renal cells [83] and the central nervous system [84]. The percentage of COX-negative fibres often correlates with disease severity and progression [85]. The biochemical defect develops within individual muscle fibres independent of the status of adjacent myofibres, likely due to the clonal expansion of mutant mtDNA, and appears to be an intrinsic property of the intracellular mitochondrial genome, largely independent of the nuclear genome [86,87]. Heteroplasmic mtDNA mutations are unevenly distributed along longitudinal syncytial muscle fibres, such that adjacent segmental sections can have widely varying amounts of mutant mtDNA [31]. Defined regions of COX deficiency have been documented in biopsies from patients with CPEO and MERRF [88,89]. There can be a striking variation in the length of COX-negative segments in the same biopsy, and the same muscle fibre can contain multiple non-contiguous COX-negative segments [60]. The latter suggests that the COX deficit may appear at multiple sites along a diseased fibre, with the length of the COX-negative segments expanding over time to coalesce with other COX-negative segments [90]. This could be due to continuous mtDNA replication in syncytial myofibres leading to changing proportions of mutant mtDNA through random intracellular genetic drift, and its lengthwise propagation over time [87]. Long COX-negative segments may eventually cause fibre atrophy, but do not lead to acute myonecrosis. It is also apparent that in biopsies from patients with various mtDNA mutations, a spectrum of deficiency exists with presence of fibres that show staining properties between completely COX-negative (blue) and COX-positive (brown) fibres, so called COX-intermediate fibres. COX-intermediate fibres, in part, represent the transition zones between COX-positive and COX-negative segments [91]. A significant difference was observed between COX-intermediate fibres and COX-positive as well as COX-negative fibres for mutant mtDNA, even more significant for wild-type mtDNA, but not for the total mtDNA copy number, suggesting that it is the wild-type mtDNA that is the critical determinant in determining the COX activity status [92,93]. The prevalence of RRF and COX-negative fibres may vary in biopsies depending on the genotype. Frequencies of RRF and COX-negative fibres are reported to be lower in MELAS and MERRF due to mtDNA point mutations than in CPEO due to mtDNA deletion, and are usually absent in LHON due to mtDNA point mutations [94,95,96]. COX-negative fibres have been noted to occur more frequently than RRF in CPEO patients associated with mtDNA point mutations and single deletions, and multiple mtDNA deletions due to *POLG1* mutations [97]. Levels of mtDNA heteroplasmy appear to directly correlate with the frequencies of RRF and COX-negative fibres [98]. In patients with primary mtDNA mutations, despite high levels of mutant mtDNA genomes in mature muscle, myogenic progenitor satellite cells have low to undetectable levels of the causative mutation [85,99,100]. Resistance exercise strength training in a group of mitochondrial myopathy patients due to a single large mtDNA deletion led to improved muscle strength, exercise induced necrosis and regeneration, increased numbers of NCAM+ satellite cells, and increased oxidative capacity including decreased percentage of COX-negative fibres and increased percentage of COX-intermediate fibres. This likely reflects a satellite cell-derived genetic drift in favour of wild-type mitochondrial genotype [91]. Taken together, it appears that, in addition to the absolute number of COX-negative fibres, the length of COX-negative segments, as well as COX-intermediate fibres, are important phenotypes to assess mitochondrial disease severity, progression and the effects of therapeutic interventions on mtDNA mutation levels and biochemical activities. 

### 5.3. SDH Deficiency

Isolated CII deficiency is a rare Mendelian mitochondrial disease due to autosomal recessive mutations in the nuclear-encoded structural sub-units and assembly factor genes of CII (*SDHA*, *SDHB*, *SDHD*, and *SDHAF1*) [101,102,103,104]. Most reported cases are of early onset, presenting with Leigh syndrome, cardiomyopathy, leukodystrophy or encephalomyopathy, with the exception of autosomal dominant mutation in *SDHA* presenting with late onset optic atrophy, ataxia and myopathy [105]. Biochemical measurement of CII in muscle is the most reliable means of diagnosis, with levels reduced to 50% or greater compared to reference mean levels. Histochemically a diffuse reduction in SDH staining with normal COX staining is demonstrable [102,106]. CII deficiency with histochemically demonstrable diffuse reduction in SDH staining in skeletal muscle, but sparing of intramuscular blood vessels is reported with autosomal recessive mutations in *ISCU* encoding for iron sulphur cluster scaffold protein, presenting with myopathy, exercise intolerance and lactic acidosis. Additional features include increased iron deposition in mitochondria and aconitase deficiency [107,108].

## 6. Associated Pathological Features 

Muscle biopsy may appear histologically normal, e.g., in patients with CI deficiency due to recessive mutations in nuclear-encoded subunits, in patients with mild RC defects, or early on in the disease course. Even in cases with heteroplasmic mtDNA mutations, there may be little abnormality apart from the presence of canonical features. Myopathic changes such as increased fibre-size variation and internal nucleation when present are typically of mild-to-moderate severity. Inflammation is absent, and necrosis and regeneration are not seen, except in mitochondrial myopathies presenting with rhabdomyolysis. Rhabdomyolysis has been associated with mutations in *CoQ2*, mtDNA encoded CIV subunit genes (*MT-CO1*, *MT-CO2*, *MT-CO3*), and tRNA genes (*MTT1*, *MT-TL1* m.3243 A > G MELAS mutation) [109,110,111,112,113,114,115,116,117,118,119,120]. Even late in the disease course, overtly dystrophic features with necrosis, fibrosis and fatty infiltration are not seen, with the exception of *TK2*-related myopathic form of mtDNA depletion syndrome [121,122]. Variable slow/type I fibre predominance and fast/type II atrophy may be present. Increased lipid may be present in fibres with or without ragged red change and COX deficiency, e.g., in KSS and PEO due to mtDNA rearrangements [21], mtDNA depletion syndrome due to mutations in *TK2*, *RRM2B*, *SUCLA2* and *SUCLG1*, and *CoQ2* [31,56,123,124,125]. Secondary carnitine deficiency with lipid storage can occur in patients with primary RCE defects [126]. The lipid storage is generally less florid when compared to primary lipid storage myopathies with massive lipidosis (primary carnitine deficiency, neutral lipid storage disease and multiple acyl-coA dehydrogenase deficiency) [127], and the presence of RRF and/or COX-negative fibres is not typically seen in the latter, although rare exceptions are reported [126]. The distinction between primary mtD and primary lipid myopathy is not possible based on muscle pathology alone, particularly in the absence of canonical mitochondrial pathology, and mild or inconstant lipidosis in the biopsy. 

## 7. Myopathology in Novel Mitochondrial Diseases

Recessive loss-of-function mutations in *CHKB* that encodes choline kinase β, an enzyme that catalyses the first de-novo biosynthetic step of phosphatidylcholine, the most abundant mitochondrial membrane phospholipid that is formed through a pathway within the mitochondria-associated endoplasmic reticulum membrane (MAM), cause a congenital muscular dystrophy with raised serum CK, severe intellectual disability with skeletal and cardiac muscle involvement, and characteristic biopsy appearances that include enlarged mitochondria at the periphery, and loss of mitochondria in the centres of myofibres, probably as a result of elimination through mitophagy and compensatory enlargement [128]. The relationship between phospholipid and mitochondrial abnormalities could be mediated via the MAM, as several proteins involved in mitochondrial dynamics are an integral part of MAM, and MAM dysfunction may mediate increase in size and intracellular displacement of mitochondria [31,129,130]. In most cases, mild dystrophic changes are consistently present in biopsies. There is variable biochemical RCE deficiency. Muscle choline kinase activity and phosphatidylcholine content are markedly reduced with aberrant remodelling of phosphatidylcholine. Loss-of-function mutations in *MICU1*, a regulator of the inner mitochondrial complex MCU, responsible for regulating mitochondrial CA^2+^ uptake and preserving normal mitochondrial CA^2+^ concentration are reported to cause a childhood-onset disease with raised CK, relatively static proximal myopathy, variable CNS involvement, and distinctive biopsy features including preserved fibre typing, mild central nucleation, mini-cores and clustered regeneration. Biochemical or histochemical RCE defects are not yet reported. There is significant loss of *MICU1* mRNA and protein in muscle, with dysfunctional mitochondrial CA^2+^ uptake in fibroblasts resulting in CA^2+^-induced fragmentation of mitochondrial networks [131]. More recently, dominant heterozygous and recessive compound heterozygous loss-of-function mutations in *MSTO1* have been characterised by whole exome sequencing in patients presenting with a multisystem disease characterized mainly by myopathy, ataxia, endocrine dysfunction and psychiatric symptoms. Serum CK ranges from normal to moderate elevation, and biopsies show myopathic or dystrophic changes, without histochemical and biochemical RC OXPHOS deficiency. Reduced levels of MSTO1 mRNA and protein in fibroblasts is associated with abnormalities of the mitochondrial network including fragmentation, aggregation, decreased network continuity and fusion activity, pointing to a putative role for *MSTO1* in mitochondrial morphogenesis by regulating mitochondrial fusion, and loss-of-function mutations linked to a multisystem mitochondrial disease [132,133]. Defects in *CHKB*, *MICU1* and *MSTO1* are examples of novel pathomechanisms and overlapping clinicopathological features involving muscular dystrophy, lipid metabolism, congenital myopathy and mitochondrial biology, with unique and recognizable muscle pathology signatures in absence of primary OXPHOS defects involving the RCE.

## 8. Vascular Pathology

Mitochondrial vasculopathy can manifest in large blood vessels (macroangiopathy) or small blood vessels (microangiopathy) including small arteries, arterioles, venules and capillaries. The clinical manifestations of macroangiopathy include premature atherosclerosis, arterial ectasia, vascular malformation, spontaneous rupture and reduced flow-mediated vasodilation [134]. In a 15-year old girl with the m.3243 A > G mutation, fatal spontaneous rupture of the aorta was associated with disorganisation and reduced COX staining in the vascular smooth muscle cells (VSMCs) of the aortic vasa vasora, and 85% mutation load in the arteries compared to 40% in blood lymphocytes [135]. Microangiopathy can manifest clinically as leukoencephalopathy, migraine-like headaches, stroke-like episodes or peripheral retinopathy. Careful morphological assessment in skeletal muscle or other tissues may reveal morphological abnormalities in VSMCs, pericytes or endothelial cells suggesting a subclinical microangiopathy [136]. MELAS is a multisystem mtD with predominant involvement of the brain, skeletal muscle and endocrine organs [137]. Unique to MELAS, particularly in association with the m.3243 A > G mutation, are transient stroke-like episodes due to lesions in the temporal and occipital lobes. Histologically, these lesions resemble true infarcts in that they are pan-necrotic and demonstrate profound neuronal loss, microvacuolation, gliosis and eosinophilic change in surviving neurons, but their topographic distribution does not follow major vascular territories or their watershed [138]. However, the presence of a microangiopathy, both within the lesions and in extra-CNS tissue like skeletal muscle, has long been recognised, manifesting as strongly SDH reactive vessels (SSVs) containing increased mtDNA copy number and ultrastructurally enlarged mitochondria. Similar SSVs can be found in MERRF, but angiopathy is less prevalent. Similar to RRF, SSVs in MERRF are typically COX-negative, whereas in MELAS, they are COX-positive [57,58]. It is postulated that the absolute amount of COX in SSVs in MELAS due to compensatory proliferation is far greater than normal [58]. As COX binds to nitric oxide, a key molecular signal for vasodilation, supernormal COX levels in these vessels titrate out nitric oxide, preventing cerebral vasodilation and triggering the stroke-like episodes [139,140]. The microangiopathy is not restricted to m.3243 A > G MELAS patients, but also documented in patients with m.8344 A > G, and autosomal recessive *POLG* mutations [138]. In these patients, multiple ischaemic stroke-like lesions in the cerebellar cortex were associated with microvascular abnormalities including loss of VSMCs and endothelial cells, evidence of blood-brain-barrier breakdown with plasma protein extravasation and loss of endothelial tight junctions, with accompanying high heteroplasmy levels of mutated mtDNA in the vessel wall. Despite clear evidence of a structurally damaged or altered microvasculature in association with vascular COX-deficiency, precisely how these deficiencies lead to the cerebral vascular events is not fully understood. It is also not known if a more generalised sub-clinical microangiopathy is present in mtD with diverse genetic backgrounds. In patients with mitochondrial myopathy, muscle capillary growth was increased as a result of impaired OXPHOS by a hypoxia-independent mechanism, promoting increased blood flow to respiration-incompetent muscles and a mismatch between systemic oxygen delivery and oxygen utilization during cycle exercise. The capillary area was greatest in patients with more severe oxidative deficits, and twice higher around fibres with oxidative defects compared to fibres with preserved oxidative function [141]. Vascular proliferation is a characteristic pathological feature of Leigh’s encephalopathy due to a variety of mitochondrial defects causing severe OXPHOS deficits in the developing CNS [142]. Therefore, capillary proliferation driven by impaired OXPHOS may be a common consequence of mtD in highly oxidative tissues.

## 9. Ultrastructure: Pathological Features and Role in Diagnostics

A range of morphological alterations has been historically documented in patients with mitochondrial myopathy with transmission electron microscopy (TEM). These include excessive numbers of mitochondria in subsarcolemmal and intermyofibrillar locations; variation in size and shape including bizarre forms, excessively large size or length exceeding 3–4 sarcomeres; abnormalities of cristae including deficient cristae, abnormal stacking or whorling; a total absence of cristae with an amorphous granular substance replacing the cristal space; electron-dense granules; and paracrystalline structures with regular geometric periodicity [21]. Of all features, paracrystalline structures are regarded as the most pathognomonic, and are frequently present in RRF. Paracrystalline structures represent mitochondrial creatine kinase crystal formation due to upregulated activity in an attempt to compensate for the energy deficit [22]. However, these morphological changes lack specificity and may be seen in a range of myopathic and dystrophic conditions. They rarely ever provide clues to the underlying biochemical and/or genetic defect [14,20,21,22,143]. Simplification of cristae with accumulation of homogenous material is apparently a specific change seen in mtDNA depletion syndrome [144]. In adult biopsies with normal light microscopic findings, ultrastructural examination is unlikely to provide additional evidence of disease. Based on a small series of five patients, it has been suggested that the earliest ultrastructural changes in infants are often noted in endothelial cells of intramuscular blood vessels even when the myofibres themselves do not show histological or ultrastructural abnormalities [145]. Given the overall lack of specificity and the time and expense involved, the routine application of electron microscopy in the investigation of suspected mtD is questionable, particularly in the era of advanced molecular diagnostics. A recent ultrastructural study combining TEM with serial block face scanning EM (SBF-SEM) and 3D reconstruction techniques has reported features not previously described in patients with mtD include linearisation and angular arrangement of cristae, localised membrane distension, nanotunnels, and donut-shaped mitochondria. Systematic assessment of mitochondrial morphology using quantitative EM methodologies sensitive mitochondrial size, shape, and branching complexity and particularly three-dimensional reconstruction methods such as serial block face (SBF-SEM) and focused-ion beam (FIB-SEM), could be used in the future to ascertain the role of structural remodelling in certain mitochondrial and other musculoskeletal diseases [146].

## 10. Secondary Mitochondrial Abnormalities

Neither presence of RRF nor focal COX deficiency is entirely specific for primary mtD. Similar changes may be seen in skeletal muscle in the context of ageing, and in a range of genetic and acquired disorders. These include infantile Pompe disease and adult-onset acid maltase deficiency [21], occasionally in muscular dystrophies such as LGMD2A [147] and FSHD [148] rarely primary lipid storage myopathies [126]. Muscle biopsies of patients with inclusion body myositis (IBM) may show increased numbers of RRF and COX-negative fibres [149]. In IBM, the on-going inflammation and cytokine environment, the associated production of reactive oxygen and nitrogen species, and the associated endoplasmic reticulum stress have a role in the initiation of mitochondrial DNA damage, leading to the accumulation of clonally-expanded mtDNA deletions and respiratory deficiency, a phenomenon that is not compensated by the malfunctioning cell repair mechanisms [150]. Increased numbers of COX-deficient and SDH-positive fibres within atrophic perifascicular zones are a common feature in dermatomyositis [151]. Histochemical and biochemical OXPHOS dysfunction can be induced by the toxic effects of a range of drugs on mitochondrial respiration, including antiretroviral agents and statins [152], antiepileptics such as valproate, immunosuppressant and cytotoxic chemotherapeutic agents [14,20]. Accumulation of multiple mtDNA deletions and tRNA point mutations has been observed in ageing human tissues [153,154] with highest levels in post-mitotic tissues such as brain and skeletal muscle. Increased numbers of RRF and COX-negative fibres are seen in skeletal muscle of older individuals and RRF comprise an average of 0.4% of all fibres by the eighth decade [155]. The age-related mtDNA mutations appear to accumulate randomly in certain myofibre segments to very high levels resulting in focal COX deficiency [156]. Overall, the amount of mutant mtDNA is very low in ageing muscle compared to patients with mitochondrial myopathy and is unlikely to cause a clinically significant OXPHOS defect [45]. Nevertheless, late-onset mitochondrial myopathy has been documented in patients over 69 years of age with multiple mtDNA deletions and increased numbers of RRF and COX-negative fibres in biopsies, possibly representing an exaggerated form of age-related mitochondrial dysfunction [157].

## 11. Myopathology of Paediatric mtD

In contrast to adults who more often present with well-defined syndromic mtD, paediatric presentations of mtD are harder to define. Neonatal or early infantile disease onset is often associated with severe progressive encephalomyopathy, with multi-organ involvement such as cardiomyopathy, hepatopathy, and myopathic involvement suggested by hypotonia, muscle weakness, wasting and arthrogryposis [123,158,159,160]. Over 90% of paediatric patients with mtD carry mutations in their nuclear genes causing defective OXPHOS [66]. This explains the long-held observation that mosaic RRF and/or COX-negative fibres are uncommon in biopsies of these patients. RRF and/or COX-negative fibres were demonstrated in 89% of biopsies with mtDNA mutations but only in 17% of biopsies without detectable mtDNA mutations in a large series of 117 children with mtD [161]. RRF and COX-negative fibres, and increased lipid are usually present in biopsies from children with mtDNA depletion syndromes secondary to defects in nuclear gene involved in mtDNA maintenance and in the myopathic form of CoQ10 deficiency [158]. COX-deficient fibres may outnumber RRF and may be the only abnormal finding in the muscle biopsy [162]. In neonates, there may be no detectable light microscopic abnormality [163]. This suggests that the compensatory proliferative response may develop over time to form RRF. The small size of fibres in biopsies from neonates and infants may make recognition of the morphological abnormality more difficult. It has been suggested that SDH-positive subsarcolemmal mitochondrial aggregates (SSMA) representing a milder form of mitochondrial proliferation is more prevalent in paediatric mtD [12]. More than 2% SSMA in patients under 16 years has been listed as a minor diagnostic criterion [23]. The sensitivity and specificity of this marker has been questioned. Such mitochondrial proliferation was absent in 35% of paediatric patients with proven mitochondrial dysfunction [164]. In 95 patients under 16 years of age, there was no difference in the frequency of SSMA between patients with and without definite mtD. Large SSMAs were observed to be more frequent in the group with definite mtD [165]. A large-scale retrospective study evaluating factors associated with SSMA in paediatric biopsies with suspected mtD found an inverse relationship between the percentage of myofibres with SSMA and RCE deficiency. Patients with low %SSMA (≤4%) were significantly more likely to develop RCE deficiency than patients with higher %SSMA (≥10%) [166]. However, it is important to note that the morphology of mitochondrial networks changes from birth to adolescence and SSMAs appear to develop over time, even in biopsies from patients in whom a primary neuromuscular disease has been excluded. Therefore, any diagnostic cut-off must take into account the confounding effect of age, and assessment of multi-centre large-scale cohorts will be necessary to develop age-stratified SSMA cut-offs with sufficiently high sensitivity and specificity to serve as a useful histological diagnostic indicator of RC deficiency in children.

## 12. Consensus Diagnostic Criteria

To facilitate the diagnosis of mitochondrial diseases, various expert groups have proposed consensus criteria and classification systems incorporating clinical, physiological, biochemical, morphological and molecular genetic criteria. Given the overlap in morphological findings associated with primary mtD and mitochondrial abnormalities secondary to ageing and various inherited and acquired non-mitochondrial neuromuscular disorders, morphological criteria essentially involve a quantitative evaluation of RRF, COX-negative fibres and SSMA in diagnostic muscle biopsies. Varying diagnostic cut-offs have been proposed including: >2% RRF and/or >2% COX-negative fibres for individuals <50 years, or >5% COX-negative fibres for individuals > 50 years [23,167,168,169]. Occasional COX-negative fibres are regarded normal > 40 years and the proportion increases with age. Any RRF < 30 years is regarded as being suspicious to warrant investigation of mtD [169]. A major limitation of the studies used to formalise such cut-offs is the lack of standardized methodology: differing biopsy sites, differing histological techniques and differing methods of quantitative assessment [52,170,171]. Age-matched control groups included patients with chronic myopathies and myositis confounding assessment due to prevalence of secondary mitochondrial abnormalities [157]. In a post mortem study evaluating biopsies from patients with and without mtD [172], <0.1% abnormal fibres were present in controls before the fifth decade. The proportion of abnormal fibres increased with age and there were regional differences (deltoid > quadriceps). Most patients with mtD had more than 0.5% abnormal fibres. Overall, COX-negative fibres were more numerous than RRF or SDH-positive fibres and provided a sensitive measure of mitochondrial abnormality. This study brings into question the widely used 2% cut-off given that the levels of abnormal fibres in controls were well below 1%. In the absence of other neuromuscular disease, mitochondrial abnormalities in muscle biopsies below the current 2% cut-off may be significant. Similarly, in paediatric biopsies, there are studies with findings challenging the currently used 2% SSMA cut-off as a minor diagnostic criterion; these are alluded to in the preceding paragraph. Notwithstanding various formal quantitative diagnostic cut-offs, it is important to remember that normal muscle morphology, especially in children, does not exclude mtD [173].

## 13. Recent Advances in Diagnostic and Research Tools: Immunoassays, Transcriptomics and Biomarkers 

The absence of reliable histochemical assays to evaluate complex I, which is the largest and most commonly affected OXPHOS complex, as well as CIII and CV is a serious limitation to the histochemical analysis of RC defects in mtD. Catalytic deficiency of RC is most often associated with a decreased amount of the assembled complex. This fact underlies the application of immunohistochemistry as a tool for investigating RC defects [174,175,176,177,178,179]. Secondly, an ever-increasing array of highly specific monoclonal antibodies is available against components of the mitoproteome spanning the nDNA and mtDNA-encoded compartments. A severe and selective reduction of immunolabelled mtDNA encoded COXI and COXII subunits with normally labelled nDNA encoded COXIV and COXVa subunits in histochemically COX-negative fibres were observed in patients with mtDNA mutations. nDNA-encoded COXVIc immunostaining was however also reduced. This was thought to relate to the holoenzyme’s quaternary structure with close interaction between COXII and COXVIc, while other nDNA encoded subunits with preserved immunoreactivity could form stable partial complexes in absence of mtDNA encoded subunits [180]. Different patterns of subunit expression were reported in the same study in mtDNA depletion syndrome including selective and non-selective loss of mtDNA encoded CIV subunits, suggesting differences in genetic background or the disease stage. Rapid protocols have been developed for fluorescent or peroxidase labelled immunostaining of cultured fibroblasts on coverslips or as cytospins using monoclonal antibodies [179,181]. Heteroplasmic mitochondrial tRNA mutations gave a heterogeneous immunostaining pattern for CI, CIII and CIV subunits as opposed to the uniformly reduced immunostaining seen in cell lines from patients with nuclear DNA defects [181]. Normal immunostaining despite reduced histochemical/biochemical activity of the corresponding complex may be due to the subcomplexes remaining active despite failure to assemble the holoenzyme, or formation of the holoenzyme with a kinetic defect. Activity dipstick assays, a type of lateral flow immunocapture assays that measure electron transfer activity of CI and CIV were developed as rapid, accurate and reproducible tests that combined the specificity of immunocapture monoclonal antibodies with the functionality of enzyme activity assays [182]. Immunolabelling for anti-DNA antibodies has been applied as an alternative to in situ hybridization to study mtDNA localization and distribution in cells. In mtDNA depletion, cytoplasmic labelling for mtDNA is either absent or reduced while the intensity of nDNA labelling is unchanged [183]. Subunit-specific immunohistochemistry can also provide insights into developmental regulation of tissue specific expression of respiratory chain complexes and their relevance in understanding disease mechanisms. Using a combination of isoform-specific antibodies (COX6AH, COX6AL, COX7AH, and COX7AL) for protein expression studies by immunohistochemistry on sections and immunoblotting muscle homogenates in combination with gene expression profiling, Boczonadi et al. demonstrated evidence for an isoform switch of COX6A and COX7A in skeletal muscle that occurs around three months of age, but there was no causative link between the isoform switch and clinical recovery in reversible infantile respiratory chain deficiency [184]. Rocha et al. have developed a quadruple immunofluorescent technique enabling quantification of key subunits of respiratory chain CI and CIV together with an indicator of mitochondrial mass and a cell membrane marker enabling protein quantitation in large numbers of fibres [185]. This technique is also able to demonstrate distinct biochemical signatures in association with specific genotypes providing insights into molecular mechanisms. For instance in patients with the common m.3243A > G *MT-TL1* mutation it was observed that CIV deficiency occurs only after CI deficiency is already established, and the defect is smoothly graduated from the normal to deficient levels of both complexes in contrast to the polarised pattern seen in the *MT-ND1* mutation. Several recent studies have shown that fibroblast growth factor 21 (FGF21), a growth factor with pleiotropic effects on regulating lipid and glucose metabolism is upregulated in patients with mtD, mice with RC deficiency, and mice with defective muscle autophagy/mitophagy [186,187,188]. mRNA and protein levels of FGF21 were robustly increased in patients with mitochondrial myopathy or MELAS. The increased FGF21 expression was shown to be a compensatory response to RC deficiency, effecting enhanced mitochondrial function through an mTOR-YY1-PGC1α-dependent pathway in skeletal muscle [189]. The accuracy of FGF21 to correctly identify muscle-manifesting mtD appeared to be higher than conventional biomarkers in one study [186]. Kalko et al. analysed the whole transcriptome of skeletal muscle in patients with *TK2* mutations and compared it to normal muscle and muscle in other mitochondrial myopathies. Bioinformatics pathway analysis identified the tumour suppressor p53 as the regulator at the centre of a network of genes responsible for a coordinated response to *TK2* mutations including induction of growth and differentiation factor 15 (GDF15), leading to its identification as a potential novel biomarker of mitochondrial dysfunction [190]. This was soon validated in two subsequent studies. One study measured the serum levels of (GDF15) against FGF21 and other conventional biomarkers in patients with mtD and healthy controls, and showed that the area under the receiver operating characteristic curve was significantly higher for GDF15 than FGF21 and other biomarkers [191]. Another study showed that elevated levels of GDF15 and FGF21 correctly identified a greater proportion of patients with mtD than GDF15 or FGF21 alone [192].

## 14. Conclusions

Despite rapid advances in genetic technologies and the increasing use of high-throughput next-generation-sequencing (NGS) platforms in the diagnostic pipeline for patients with suspected mtD, the laboratory investigation of mtD is still complex, and muscle biopsy remains a key tool that provides tissue for diagnostic and functional studies to direct molecular genetic testing. Demonstration of histochemical mosaic COX deficiency provides crucial evidence for a heteroplasmic mtDNA disease. The pathologist must take into account developmental, ageing-associated and secondary mitochondrial changes whilst interpreting mitochondrial pathology in muscle biopsies. Optimal handling and processing of tissue maximises the diagnostic yield in biopsies. With increasing adoption of NGS platforms in diagnostic laboratories comes the challenge of functional testing to determine pathogenicity for variants of uncertain significance found with increasing frequency. In this context, it is incumbent upon pathologists to develop novel pathology tools incorporating advances in tissue multiplexing and imaging; enabling more objective and informatics-based assessment of OXPHOS deficiency to improve the diagnostic outcome in mtD; understand pathomechanisms of mitochondrial dysfunction in primary mtD as well as in other diseases; monitor mitochondrial disease progression; and serve as biological outcome measures in clinical trials. 
